# Integrating N signals and root growth: the role of nitrate transceptor NRT1.1 in auxin-mediated lateral root development

**DOI:** 10.1093/jxb/eraa243

**Published:** 2020-07-25

**Authors:** Katerina S Lay-Pruitt, Hideki Takahashi

**Affiliations:** 1 Genetics and Genome Sciences Program, Michigan State University, East Lansing, MI, USA; 2 Department of Biochemistry and Molecular Biology, Michigan State University, East Lansing, MI, USA

**Keywords:** Auxin, lateral root development, nitrate transporter, nitrogen, NRT1.1, root system architecture, signaling, transceptor

## Abstract

This article comments on:

**Maghiaoui A, Bouguyon E, Cuesta C, Perrine-Walker F, Alcon C, Krouk G, Benková E, Nacry P, Gojon A and Bach L**. 2020. The Arabidopsis NRT1.1 transceptor coordinately controls auxin biosynthesis and transport to regulate root branching in response to nitrate. Journal of Experimental Botany **71**, 4480–4494.

**Plants modify their root system architecture (RSA) to efficiently acquire nutrients from the environment. Nitrate (NO**_**3**_^**–**^ **) is an essential nutrient that elicits changes in RSA through the action of the NO**_**3**_^**–**^ **transceptor NRT1.1.** Maghiaoui *et al.* (2020) **have demonstrated that NRT1.1 modulates lateral root growth in response to NO**_**3**_^**–**^ **by regulating both auxin biosynthesis and downstream auxin transport during lateral root development.**

## Dynamic changes in RSA in response to nitrogen availability

The plant root system is highly dynamic; various external stimuli from the soil environment dictate root growth strategies to optimize uptake of water and nutrients. This spatiotemporal, structural arrangement of plant root biomass is referred to as root system architecture (RSA). RSA encompasses such traits as the primary (or embryonic) root length, the length and density of lateral (post-embryonic) roots, and the angles at which these roots grow (Osmont *et al.*, 2007). One way in which plants adjust RSA is through the action of auxins, a major class of phytohormones regulating plant growth, particularly the development of lateral root primordia (Benková *et al.*, 2003). Auxin gradients within these root tissues are coordinated by the action of auxin influx and efflux transporters (Petrášek and Friml, 2009).

RSA must have high plasticity because essential nutrients are heterogeneously distributed throughout the soil profile in patches and gradients (Giehl and von Wirén, 2014). Of these nutrients, nitrogen (N) has been of particular interest because it has a significant and complex effect on RSA. For example, N starvation has been shown to severely inhibit primary root growth and development of lateral root primordia (Araya *et al.*, 2014), but mild N deprivation induces lateral root growth as part of a ‘foraging’ mechanism used by the plant to acquire N (Krouk *et al.*, 2010; Gruber *et al.*, 2013). However, there remains a need for research to understand how N affects the underlying signaling processes—such as auxin transport—involved in governing RSA.

The main form of N that many plant species preferentially take from the soil is inorganic nitrate (NO_3_^–^) (Nacry *et al.*, 2013). In Arabidopsis, NO_3_^–^ uptake is facilitated by the NRT2 and NPF families of NO_3_^–^ transporters, which in general are known to have high and low affinity for NO_3_^–^, respectively (Kiba and Krapp, 2016). However, one key NPF member, NRT1.1 (NPF6.3), uniquely exhibits dual affinity for NO_3_^–^ and has been implicated in not only NO_3_^–^ transport but also NO_3_^–^-responsive control of root development (Tsay *et al.*, 1993; Liu and Tsay, 2003; Remans *et al.*, 2006; Krouk *et al.*, 2010; Bouguyon *et al.*, 2015). Specifically, NRT1.1 serves as a transporter and receptor (transceptor) of NO_3_^–^ (Ho *et al.*, 2009) as well as an auxin transporter in the lateral root primordia when NO_3_^–^ supply is low or absent (Krouk *et al.*, 2010; Bouguyon *et al.*, 2015; Box 1). Due to these multiple roles, research characterizing the role of NRT1.1 in regulating RSA provides critical insight into novel molecular pathways in which plants modulate root growth in response to environmental signals.

**Box 1. NRT1.1 plays multiple roles in NO_3_^–^ responses**

NRT1.1 (also referred to as NPF6.3 or CHL1) was one of the first major plasma membrane-bound NO_3_^–^ transporters in plants to be characterized (Tsay *et al.*, 1993). Although members of the NPF family typically have low affinity for NO_3_^–^, NRT1.1 uniquely exhibits dual affinity for NO_3_^–^ (Liu and Tsay, 2003). NRT1.1 is also of interest since it is one of the few characterized ‘transceptors’ in plants, a protein that may function both as a transporter and as a receptor. Not only does NRT1.1 have known function in transporting NO_3_^–^, it acts as a transceptor dephosphorylated/phosphorylated at the Thr101 residue with involvement of NO_3_^–^-responsive CIPK23, a calcineurin B-like protein-interacting protein kinase, to signal on/off gene expression of a high-affinity NO_3_^–^ transporter NRT2.1 (Ho *et al.*, 2009). This phosphorylation event is also suggested to alter the dimerization and structural flexibility of NRT1.1, which is correlated with changes in the affinity state (Sun and Zheng, 2015). NRT1.1 also acts to transport auxin under conditions of low and no NO_3_^–^ availability (Krouk *et al.*, 2010; Bouguyon *et al.*, 2015). The auxin transport activity of NRT1.1 may be attributed to the Thr101 phosphorylation status that allows this transporter to remain active with the high-affinity NO_3_^–^ transport kinetics but eliminates the low-affinity kinetics. When roots are subjected to conditions with no NO_3_^–^ supply, NRT1.1 basipetally transports auxin toward the direction away from the apex of lateral root primordia. Thus, less auxin accumulates in the meristem of developing primordia. Development of lateral root primordia will then be restricted prior to emergence from the primary root through this NRT1.1-mediated auxin transport mechanism and in conjunction with the reduction in *LAX3* and *TAR2* gene expression, as highlighted in Maghiaoui *et al.* (2020). Besides these mechanisms, NRT1.1 also signals to activate gene expression of a MADS-box transcription factor ANR1 to promote lateral root elongation in response to local NO_3_^–^ supply (Remans *et al.*, 2006). The induction of *NRT2.1* and potentially *ANR1* gene expression occurs downstream of NO_3_^–^ and NRT1.1-induced Ca^2+^ signaling, promoting the nuclear localization of the transcription factor NLP7 (Krapp *et al.* 2014; Liu *et al.* 2017; Zhang *et al.* 2019).

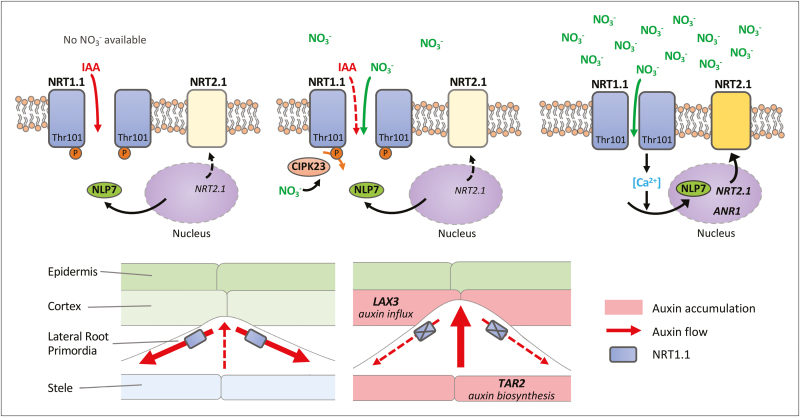

## NRT1.1 acts as an integrator of NO_3_^–^ signaling and auxin biosynthesis and transport

In their work published in this issue, Maghiaoui *et al.* (2020) have demonstrated additional roles of NRT1.1 as an integrator optimizing auxin signaling pathways, highlighting the importance of this transceptor in modulation of RSA. In order to first determine the effect of NO_3_^–^ on known families of auxin transporters, the authors analyzed the expression levels of key auxin transporters in wild-type seedlings and the *chl1-5* mutant line lacking *NRT1.1* expression by growing them with or without NO_3_^–^ supplementation. Their results show that gene and protein expression of various PIN, ABCB, and AUX/LAX auxin transporters are N responsive but not in an NO_3_^–^-specific or NRT1.1-dependent manner, as re-supplementation of glutamine restores their expression in the *chl1-5* mutant.

However, the one exception to these trends was LAX3, an auxin influx transporter that imports auxin into cortical cells overlying the lateral root primordia, which leads to loosening of the cell wall and allows the primordia to emerge as they develop (Swarup *et al.*, 2008). *LAX3* gene and protein expression as well as gene expression of *LBD29*, an upstream regulator of *LAX3* (Porco *et al.*, 2016), were found to be repressed in the wild type but not in the *chl1-5* mutant in the absence of NO_3_^–^ supplementation. Additionally, there was no recovery of *LAX3* expression by glutamine. Further evidence indicates that a key auxin biosynthetic gene, *TAR2*, is also suppressed by NRT1.1 under NO_3_^–^ deprivation. TAR2 had previously been implicated in controlling root development as mild N deficiency induces TAR2 expression, which in turn promotes auxin accumulation and lateral root emergence for N foraging; however, this function was not characterized in relation to NRT1.1 (Ma *et al.*, 2014). Maghiaoui *et al.* (2020) thus propose that repression of *LAX3* and *TAR2* gene expression occurs through a putative upstream signaling module involving NRT1.1, and this prevents the growth of lateral root primordia under low NO_3_^–^ availability. These results also emphasize how NO_3_^–^ specifically acts to regulate auxin transport and lateral root development, supporting previous findings from Krouk *et al.* (2010).

In summary, Maghiaoui *et al.* (2020) demonstrate that NRT1.1 acts as an integrator for NO_3_^–^-derived signals on two sides of auxin signaling, biosynthesis and transport, during the development of the lateral root primordia. These results add to our knowledge of the NO_3_^–^ transceptor NRT1.1 and open up questions about NO_3_^–^ as a specific signaling molecule modulating auxin distribution in a multifaceted manner—primarily affecting basipetal auxin transport within the primordia, and additionally required for controlling gene expression of an auxin influx transporter at overlaying cortical cells and a key auxin biosynthetic gene in the vasculature (Box 1). It remains to be investigated how NRT1.1 transmits the signal and impacts on auxin signaling in other aspects of RSA.

## Future perspectives: the impact of N and auxin on RSA

There are multiple avenues for further research that will deepen our understanding of RSA development in response to inorganic N in soils. Plants have the capacity to take up and utilize N in the form of ammonium (NH_4_^+^) in addition to NO_3_^–^ (Hachiya and Sakakibara, 2017). NH_4_^+^ uptake is mediated by the AMT family of transporters (Yuan *et al.*, 2007). NH_4_^+^ availability has been shown to elicit changes in RSA in a distinct manner, promoting higher order branching of lateral roots in contrast to NO_3_^–^-driven mechanisms, which may reflect the differences between accumulation of these N sources within the soil profile (Lima *et al.*, 2010; Kiba and Krapp, 2016). Thus, NH_4_^+^ could also be exerting changes in auxin transport or biosynthesis pathways to modulate RSA. Additionally, there is evidence that NRT1.1 plays a role in Arabidopsis response to NH_4_^+^ toxicity, suggesting that NO_3_^–^ and NH_4_^+^ response mechanisms may partially overlap (Jian *et al.*, 2018). The interaction of these two forms of inorganic N and how they regulate lateral root development could be a promising area for further research.

Another direction of inquiry would be to determine whether NRT1.1-dependent auxin signaling differs depending on local or homogenous NO_3_^–^ availability. In Arabidopsis, NRT1.1 has been implicated in lateral root proliferation in response to local NO_3_^–^ supply (Remans *et al.*, 2006; Mounier *et al.*, 2014). It would be interesting to see whether the pathways involving LAX3 and TAR2 elucidated in the study by Maghiaoui *et al.* (2020) are conserved when plants are experiencing local supplementation of NO_3_^–^ and how this could affect development of primordia located in distal parts of the root. A subsequent extension of this research would be to study the potential impact of this pathway on development of lateral root primordia with relevance to higher order branching of lateral roots, as has been observed with a local supply of NH_4_^+^ (Lima *et al.*, 2010).

This research provides novel insight into regulation of auxin transport, biosynthesis, and signaling pathways modulating RSA, and further highlights a key question in understanding the integration of plant development and the NO_3_^–^ environment: what other types of developmental processes could be regulated by proteins with versatile functions like the NO_3_^–^ transceptor NRT1.1? The mechanisms highlighted in this study may also have interesting implications for other plant species beyond dicots, for example OsNRT1.1B in *Oryza sativa*, given its essential role in increasing the N use efficiency (Hu *et al.*, 2015). Extension of these concepts into an agriculturally relevant plant species will allow researchers to bridge basic and applied research approaches.
